# Naoxueshu relieves hematoma after clot removal in acute spontaneous intracerebral hemorrhage

**DOI:** 10.1002/brb3.1957

**Published:** 2020-12-04

**Authors:** Juexian Song, Yuting Nie, Pingping Wang, Huiqiang Lu, Li Gao

**Affiliations:** ^1^ Department of Neurology Xuanwu Hospital Capital Medical University Beijing China; ^2^ The Key Laboratory of development biology College of Life Sciences Jinggangshan University Jinggangshan China

**Keywords:** hematoma, intracerebral hemorrhage, surgery, traditional Chinese medicine

## Abstract

**Objectives:**

Surgical treatment is expected to remove clot immediately in acute spontaneous intracerebral hemorrhage (SICH) patients. The aim of this study was to evaluate whether Naoxueshu could enhance the efficacy of clot removal surgery in acute SICH patients.

**Methods:**

One hundred twenty patients who had been diagnosed as SICH according to neuroimaging were enrolled in this study. They received craniotomy, decompressive craniectomy, or minimally invasive surgical evacuation as appropriate and then were randomized into two groups: the Naoxueshu group (NXS group, *n* = 60) and the control group (*n* = 60). All the patients received standard medical management while patients in NXS group also took Naoxueshu oral liquid 10 ml with three times a day for seven consecutive days. The primary outcome was the 7‐day hematoma volume and secondary outcomes were 7‐day National Institutes of Health Stroke Scale (NIHSS) score and 7‐day cerebral edema score.

**Results:**

After clot removal surgery, hematoma volume in NXS group (9.5 ± 8.0) was significantly decreased than that in Control group (21.3 ± 22.9, *p* < .0001) 7 days after surgery. Moreover, cerebral edema was also relieved after 7‐day's Naoxueshu treatment (2.5 ± 0.9 vs. 2.9 ± 0.7, *p* = .043). Since patients in NXS group had worse baseline NIHSS score (17.2 ± 8.1 vs. 13.7 ± 10.1, *p* = .039), it was reasonable to conclude that Naoxueshu treatment could improve patients’ neurological function because 7‐day NIHSS score of the two groups was similar.

**Conclusion:**

Naoxueshu oral liquid could relieve hematoma volume and cerebral edema after clot removal surgery in acute SICH patients. Moreover, it had the potential to improve patients’ short‐term neurological function.

## Graphical abstract

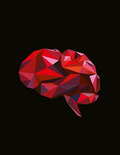

## 1 INTRODUCTION

Spontaneous, nontraumatic intracerebral hemorrhage (ICH) remains one of the most devastating neurologic diseases. ICH accounts for 6.5%–19.6% of all strokes worldwide (Carolei et al., 1997; Feigin et al., 2003) and 30‐day mortality rates have been reported to be approximately 40%–50% (Broderick et al., 1993; Hemphill et al., 2001). Unlike ischemic stroke, spontaneous intracerebral hemorrhage (SICH) has no disease‐modifying treatment and the mainstay of treatment for SICH is limited to reversal of coagulopathy and control of hypertension. According to the latest AHA/ASA guidelines for the management of SICH, only patients with deteriorating neurologically cerebellar hemorrhage or brainstem compression should undergo surgical removal of the hemorrhage as soon as possible (Class I; Level of Evidence B) (Hemphill et al., 2015). Some randomized controlled clinical trials (RCT) have indicated that minimally invasive approaches to clot evacuation in SICH are effective (Hanley et al., 2017; Vespa et al., 2016; Wang et al., 2009). However, according to the latest MISTIE III results, minimally invasive surgery with thrombolysis could improve 7‐day mortality rate but not a good response 365 days after ICH (Hanley et al., 2019). So the role of clot removal surgery for most patients with SICH remains controversial and exploring effective neuroprotective drugs to enhance the efficacy of SICH surgery is an urgent mission.

Because of the disappointments in western medicines in patients with stroke, recently attention has turned toward traditional Chinese medicine (TCM). TCM has been widely used for ischemic stroke for thousands of years in China. Naoxueshu oral liquid is a TCM patent drug including leech, astragalus root, Rhizome Chuanxiong calamus, and Achyranthes. According to the TCM theory that “Qi is the commander of blood; blood is the source of Qi” (Chen et al., 2014), Naoxueshu replenish Qi, activate blood circulation and remove blood stasis. So in Chinese clinical practice, it is mainly used for hemorrhagic stroke in patients with Qi deficiency and blood stasis (Chen et al., 2014). A recent systematic review and Meta‐Analysis showed that the application of Naoxueshu oral liquid alone or combined with other drugs were effective in the treatment of ICH, especially hypertensive ICH (Wu et al., 2017). Whether it could be useful in SICH patients who had received clot removal surgery was still unknown. Therefore, the aim of this study was to explore the efficiency of Naoxueshu oral liquid after clot removal surgery in SICH patients.

## 2 METHODS

This study was a random, multicenter, open‐label, prospective study with two groups. One hundred and twenty SICH patients were enrolled consecutively and randomly assigned to the Naoxueshu group (NXS group, *n* = 60) and the control group (*n* = 60). All the patients or family members of the study were informed, and the written consent of the patients was obtained. This study was approved by the Ethics Committee of Xuanwu Hospital, Capital Medical University.

### 2.1 Patients

One hundred and twenty SICH patients were recruited consecutively from December 2016 to August 2018 in 6 hospitals in Beijing, China. Diagnosis of SICH was confirmed by neuroimaging such as magnetic resonance imaging (MRI) and computer tomography (CT). Other inclusion criteria were (a) in accordance with the diagnostic criteria of SICH approved by AHA/ASA guideline (Hemphill et al., 2015); (b) 18–75 years old; (c) SICH within 7 days (including 7 days) (d) NIHSS score between 2 and 24. Patients with loss of consciousness, incomplete hepatic or renal function, or severe psychotic disease were excluded. The baseline characteristics of the study subjects are shown in Table 1.

**TABLE 1:** Baseline characteristics of the patients

Characteristics	NXS group (*n* = 60)	Control group (*n* = 60)	*p* value
Age (years)	55.1 ± 13.4	58.9 ± 11.3	.098
Male sex	39 (65.0%)	39 (65.0%)	1.000
Heart rate (*n*/min)	82.6 ± 15.1	80.1 ± 18.7	.570
Temperature (℃)	36.8 ± 0.6	36.9 ± 0.9	.562
Systolic pressure (mmHg)	163.8 ± 24.2	153.2 ± 23.4	.017
Diastolic pressure (mmHg)	98.0 ± 19.8	86.9 ± 15.8	.001
Cause of SICH			
Hypertension	35 (58.3%)	38 (63.3%)	.497
Intracranial aneurysm or AVM	23 (38.3%)	18 (30.0%)	
Others	2 (3.3%)	4 (6.7%)	
Baseline NIHSS score	17.2 ± 8.1	13.7 ± 10.1	.039
Hematoma volume (ml)	34.2 ± 18.5	31.1 ± 28.0	.473
Baseline cerebral edema score	3.1 ± 1.0	3.0 ± 0.9	.428

Abbreviations: AVM, arteriovenous malformation; NIHSS, National Institutes of Health Stroke Scale; SICH, spontaneous intracerebral hemorrhage.

### 2.2 Therapeutic methods

All the patients received early clot removal surgery (craniotomy, decompressive craniectomy or minimally invasive surgical evacuation as appropriate) conducted by experienced neurologists once SICH had been diagnosed. All the patients in both groups had standard medical management including: (a) 20% mannitol of dehydration treatment; (b) general monitoring and nursing care; (c) blood pressure control; (d) supportive therapy. Only patients in NXS group took Naoxueshu oral liquid 10 ml with three times a day from the first day after surgery for seven consecutive days.

### 2.3 Study outcomes

The primary outcome was the 7‐day hematoma volume and secondary outcomes were 7‐day National Institutes of Health Stroke Scale (NIHSS) score and 7‐day cerebral edema score. Other observational factors included results of blood routine examination, coagulation function, liver function, and renal function.

Hematoma volume (ml) = *π*/6 × length (cm) × width (cm) × high (cm) (Song et al., 2018), and it was determined by CT scanning (Siemens 64‐slice CT machine, section thickness, 5 mm; gap, 5 mm; pitch, 1; tube current, 304mA; and voltage, 120 kV).

Cerebral edema severity was determined by four grades: (1) No cerebral edema; (2) Formation of perihematoma edema zone; (3) Manifestation of grade 2 plus ventricular compression; (4) Midline shift in addition to manifestation of grade 2 and grade 3.

### 2.4 Statistical analysis

All statistical analyses were conducted by SPSS 19.0 (SPSS Inc.). Continuous variables were presented as means ± standard deviations (*SD*), and they were evaluated with *t* test. The category variables were presented as numbers and frequencies, and they were compared with the chi‐squared test or the *Fisher's exact* test as appropriate. Intra group baseline and 7‐day factors were analyzed by paired‐samples *t* test, while between group factors comparison was conducted by independent samples *t* test. The *p* value <.05 was considered to statistically significant.

## 3 RESULTS

### 3.1 Outcomes comparison

Although all the patients received immediate clot removal surgery, 7 days after surgery, hematoma volume in NXS group (9.5 ± 8.0) was significantly decreased than that in Control group (21.3 ± 22.9, *p* < .0001, Table 2). Moreover, cerebral edema was also relieved after 7‐day Naoxueshu treatment (2.5 ± 0.9 vs. 2.9 ± 0.7, *p* = .043, Table 2). Since patients in NXS group had worse baseline NIHSS score (17.2 ± 8.1 vs. 13.7 ± 10.1, *p* = .039, Table 1), it was reasonable to conclude that Naoxueshu treatment could improve patients’ neurological function because 7‐day NIHSS score of the 2 groups was similar (10.3 ± 7.0 vs. 10.3 ± 9.2, *p* = .969, Table 2).

**TABLE 2:** Outcome measures of the two groups

Outcomes	NXS group (*n* = 60)	Control group (*n* = 60)	*p* value
Primary outcome			
7‐day hematoma volume (ml)	9.5 ± 8.0	21.3 ± 22.9	<.0001
Secondary outcomes			
7‐day NIHSS score	10.3 ± 7.0	10.3 ± 9.2	.969
7‐day cerebral edema score	2.5 ± 0.9	2.9 ± 0.7	.043

Abbreviation: NIHSS, National Institutes of Health Stroke Scale.

### 3.2 Other observational factors comparison

The level of fibrinogen (Fib) was elevated significantly in both groups 7 days after SICH occurrence (both *p* < .0001, Table 3). However, there was no statistical difference in 7‐day Fib between the two groups (*p* = .329, Table 4). In other words, Naoxueshu treatment did not affect Fib level in SICH patients. No statistical significance was observed in 7‐day results comparison of blood routine examination, coagulation function, liver function, or renal function (Table 4).

**TABLE 3:** Intra group comparison of other observational factors

Factors	NXS group (*n* = 60)		*p* value	Control group (*n* = 60)		*p* value
	Baseline data	7‐day data		Baseline data	7‐day data	
WBC (×10^9^/L)	12.0 ± 4.7	10.8 ± 3.4	.069	11.2 ± 5.3	9.7 ± 2.7	.055
HGB (g/L)	137.4 ± 22.0	133.6 ± 19.4	.321	137.2 ± 24.2	131.0 ± 20.8	.139
PLT (×10^9^/L)	207.1 ± 98.2	218.0 ± 80.8	.514	223.0 ± 81.1	217.6 ± 62.8	.8684
APTT (s)	28.9 ± 6.8	29.5 ± 6.1	.629	32.4 ± 5.5	31.13 ± 6.5	.436
PT (s)	12.5 ± 2.2	12.8 ± 2.1	.370	13.9 ± 2.0	13.6 ± 1.4	.505
Fib (g/L)	3.3 ± 1.8	4.6 ± 2.0	<.0001	3.8 ± 1.3	5.0 ± 1.8	<.0001
ALT (U/L)	28.1 ± 18.9	37.4 ± 32.7	.065	24.3 ± 13.4	29.0 ± 17.7	.107
AST (U/L)	29.2 ± 13.4	34.0 ± 19.9	.129	26.8 ± 11.7	31.6 ± 26.9	.205
Cr (μmol/L)	60.2 ± 13.2	58.1 ± 19.3	.497	63.6 ± 21.6	56.4 ± 23.2	.081
BUN (mmol/L)	5.8 ± 2.5	6.3 ± 2.4	.341	5.6 ± 3.1	6.3 ± 2.6	.144

Abbreviations: ALT, alanine aminotransferase; APTT, activated partial thromboplastin time; AST, aspartate aminotransferase; BUN, blood urea nitrogen; Cr, creatinine; Fib, fibrinogen; Hgb, hemoglobin; PLT, Platelet; PT, prothrombin time; WBC, white blood cell.

**TABLE 4:** Between group comparison of other observational factors

Factors	NXS group (*n* = 60)	Control group (*n* = 60)	*p* value
WBC (×10^9^/L)	10.8 ± 3.4	9.7 ± 2.7	.187
Hgb (g/L)	133.6 ± 19.4	131.0 ± 20.8	.242
PLT (×10^9^/L)	218.0 ± 80.8	249.1 ± 242.3	.570
APTT (s)	29.5 ± 6.1	31.13 ± 6.5	.175
PT (s)	12.8 ± 2.1	13.6 ± 1.4	.065
Fib (g/L)	4.6 ± 2.0	5.0 ± 1.8	.329
ALT (U/L)	37.4 ± 32.7	29.0 ± 17.7	.088
AST (U/L)	34.0 ± 19.9	31.6 ± 26.9	.672
Cr (μmol/L)	58.1 ± 19.3	56.4 ± 23.2	.665
BUN (mmol/L)	6.3 ± 2.4	6.3 ± 2.6	.986

Abbreviations: ALT, alanine aminotransferase; APTT, activated partial thromboplastin time; AST, aspartate aminotransferase; BUN, blood urea nitrogen; Cr, creatinine; Fib, fibrinogen; Hgb, hemoglobin; PLT, Platelet; PT, prothrombin time; WBC, white blood cell.

## 4 DISCUSSION

The results of our study showed that Naoxueshu oral liquid is effective in reducing hematoma volume and cerebral edema after clot removal surgery in SICH.

In recent decades, there is great interest among researchers in studying efficacy of TCM in stroke patients, especially in China. Naoxueshu oral liquid was extracted from astragalus root, leech, calamus, Achyranthes, and Rhizoma Chuanxiong. Astragalus was demonstrated to reduce brain damage of neuronal mitochondria after hemorrhage, inhibit neuronal apoptosis, and promote the recovery of neurological function. Leech has a cerebral protective effect of anticoagulation, inhibiting platelet aggregation, and improving blood rheology (Cai et al., 2014; Dong et al., 2016). Wu and his colleagues (Wu et al., 2017) conducted a systematic review and Meta‐Analysis of 14 studies (1,606 patients) and found that Naoxueshu oral liquid in the treatment of acute cerebral hemorrhage had more obvious effects than conventional medical treatment. However, because the active components of TCM often have not been specified and measured precisely, the efficacy of Naoxueshu and other neuroprotective TCM is still under debate. Efforts to reveal the molecular mechanism of TCM in stroke have been done in some animal models. Qi‐supplementing therapy could up‐regulate the expressions of vascular endothelial growth factor (VEGF) and its receptors (Flk‐1 and Flt‐1) (Zhang et al., 2007), improve the effect on lipid peroxidation (Qiang et al., 2000), up‐regulate heme oxygenase 1 (HO 1) expression (Tao et al., 2002), and increase neural stem cells (Tang et al., 2004), thus improving the recovery of ICH rats. As a kind of Qi‐supplementing therapy, the concept of this patent drug, Naoxueshu, is to inhibit the coagulation process, promote fibrinolysis, expand blood vessels, and improve microcirculation (Kang et al., 2015). The beneficial effect of hematoma absorption of Naoxueshu oral liquid in SICH patients could at least partly be explained by these mechanisms.

Clot removal surgery is expected to prevent brain herniation, reduce intracranial pressure, decrease mass effect of the hematoma on surrounding tissue, and eliminate the cellular toxicity of blood products. However, early hematoma removal has not been shown to be beneficial in two large randomized trials (Mendelow et al., 2005, 2013). Similarly, neither CLEAR III trial (Hanley et al., 2017) nor MISTIE III trial (Hanley et al., 2019) was able to demonstrate improvement of functional outcomes in SICH. In our study, clot removal and standard medical management had limited efficacy in reducing hematoma volume (from 31.1 ± 28.0 ml to 21.3 ± 22.9 ml in Control group). Moreover, NIHSS score in Control group decreased insignificantly 7 days after surgery (from 13.7 ± 10.1 to 10.3 ± 9.2). All these results were in accordance with the results of the RCTs mentioned above.

The main reason for cerebral hemorrhage with high mortality and disability rates is cerebral edema after brain injury because it increases intracranial pressure and it can form severe cerebral hernia. Therefore, early intervention is important in SICH patients. Four possible factors together induce cerebral edema after ICH: (a) vasogenic factors including clot formation and contraction, decrease in hydrostatic pressure around hematoma space and plasma proteins extravasation; (b) inflammation, which leads to blood–brain barrier (BBB) disruption, (c) thrombin, which is associated with BBB disruption and cell toxicity; (d) red blood cell (RBC) lysis production like hemoglobin, which can cause increase in BBB permeability (Zheng et al., 2016). Our study demonstrated that Naoxueshu treatment could relieve cerebral edema after SICH and surgery (from 3.1 ± 1.0 points to 2.5 ± 0.9 points). That might be attributed to one component of Naoxueshu oral liquid, leech, which was proved to be effective in relieving brain edema by many modern researches (Dong et al., 2016; Li et al., 2007).

Previous study found anti‐inflammatory effects of Naoxueshu because the level of white blood cell (WBC) count was decreased more significantly in Naoxueshu treatment patients (Song et al., 2018). However, in this study, change of WBC count was not so significant. That could be explained by the short observation time (7 days in this study and 21 days in Song's study) of our study. On the other hand, we found significantly elevated Fib level in both groups, which was even higher than the result of Song's study. That is to say, release of Fib occurred immediately after SICH, and it could last for at least 21 days.

Safety is another important issue of TCM. As we know, TCM is not side‐effect free and there can be direct toxic or allergic reactions and interactions with other medicines in its use. Our results suggested that Naoxueshu did not affect SICH patients’ coagulation function, liver function, and renal function after surgery, indicating that Naoxueshu oral liquid was a safe treatment for SICH.

## 5 CONCLUSION

Naoxueshu oral liquid could relieve hematoma volume and cerebral edema safely after early surgical hematoma removal in acute SICH patients. Moreover, it had the potential to improve patients’ short‐term neurological function.

## CONFLICT OF INTEREST

There is no conflict of interest in this article.

## AUTHOR CONTRIBUTION

Juexian Song and Li Gao designed this study; Juexian Song, Yuting Nie, and Pingping Wang performed the clinical management and data collection; Huiqiang Lu performed the statistical analysis; Juexian Song drafted the manuscript and all authors reviewed, revised, and approved the final manuscript.

### Peer Review

The peer review history for this article is available at https://publons.com/publon/10.1002/brb3.1957.

## Data Availability

The original data will be available when contact the correspondence author.
